# (4,7-Diphenyl-1,10-phenanthroline-κ^2^ *N*,*N*′)diiodidomercury(II)

**DOI:** 10.1107/S160053680803081X

**Published:** 2008-09-27

**Authors:** Mohammad Yousefi, Rabin Rashidi Vahid, Vahid Amani, Mansour Arab Chamjangali, Hamid Reza Khavasi

**Affiliations:** a Islamic Azad University, Shahr-e-Rey Branch, Tehran, Iran; b Department of Chemistry, Shahrood University of Technology, Shahrood, Iran; c Department of Chemistry, Shahid Beheshti University, Tehran 1983963113, Iran

## Abstract

In the mol­ecule of the title compound, [HgI_2_(C_24_H_16_N_2_)], the Hg^II^ atom is four-coordinated in a distorted tetra­hedral configuration by two N atoms from the bidentate 4,7-diphenyl-1,10-phenanthroline and two iodide ligands. There is a π–π contact between the pyridine and phenyl rings [centroid-to-centroid distance = 4.2387 (4) Å].

## Related literature

For related literature, see: Ahmadi, Amani *et al.* (2008 ▶); Ahmadi, Kalateh, Ebadi *et al.* (2008 ▶); Ahmadi, Kalateh, Abedi *et al.* (2008 ▶); Ahmadi, Khalighi *et al.* (2008 ▶); Khalighi *et al.* (2008 ▶); Khavasi *et al.* (2008 ▶); Tadayon Pour *et al.* (2008 ▶); Yousefi, Khalighi *et al.* (2008 ▶). For related structures, see: Chen *et al.* (2006 ▶); Freire *et al.* (1999 ▶); Htoon & Ladd (1976 ▶); Yousefi, Tadayon Pour *et al.* (2008 ▶).
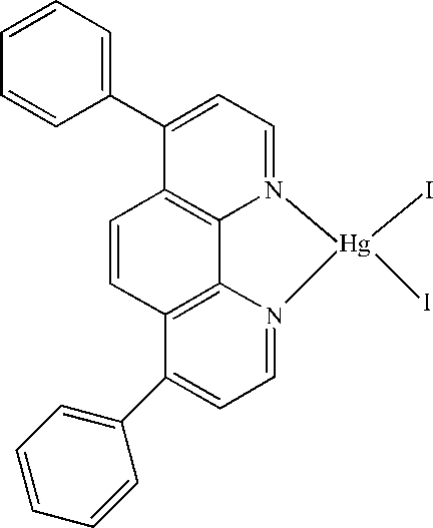


## Experimental

### 

#### Crystal data

[HgI_2_(C_24_H_16_N_2_)]
*M* *_r_* = 786.78
Monoclinic, 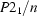

*a* = 16.673 (3) Å
*b* = 8.8964 (18) Å
*c* = 16.823 (3) Å
β = 109.26 (3)°
*V* = 2355.7 (9) Å^3^
*Z* = 4
Mo *K*α radiation
μ = 9.17 mm^−1^
*T* = 298 (2) K
0.50 × 0.48 × 0.28 mm

#### Data collection

Bruker SMART CCD area-detector diffractometer
Absorption correction: numerical shape of crystal determined optically (*X-SHAPE* and *X-RED*; Stoe & Cie, 2005 ▶)*T*_min_ = 0.016, *T*_max_ = 0.080
19129 measured reflections
6340 independent reflections
5356 reflections with *I* > 2σ(*I*)
*R*_int_ = 0.094

#### Refinement

*R*[*F* ^2^ > 2σ(*F* ^2^)] = 0.067
*wR*(*F* ^2^) = 0.183
*S* = 1.23
6340 reflections
263 parameters
H-atom parameters constrained
Δρ_max_ = 1.49 e Å^−3^
Δρ_min_ = −1.10 e Å^−3^

### 

Data collection: *SMART* (Bruker, 1998 ▶); cell refinement: *SAINT* (Bruker, 1998 ▶); data reduction: *SAINT*; program(s) used to solve structure: *SHELXTL* (Sheldrick, 2008 ▶); program(s) used to refine structure: *SHELXTL*; molecular graphics: *ORTEP-3 for Windows* (Farrugia, 1997 ▶); software used to prepare material for publication: *WinGX* (Farrugia, 1999 ▶).

## Supplementary Material

Crystal structure: contains datablocks I, global. DOI: 10.1107/S160053680803081X/hk2540sup1.cif

Structure factors: contains datablocks I. DOI: 10.1107/S160053680803081X/hk2540Isup2.hkl

Additional supplementary materials:  crystallographic information; 3D view; checkCIF report

## Figures and Tables

**Table:** 

I1—Hg1	2.6441 (8)
I2—Hg1	2.6555 (9)
N1—Hg1	2.425 (7)
N2—Hg1	2.399 (7)

**Table:** 

N2—Hg1—N1	69.4 (2)
N2—Hg1—I1	104.08 (17)
N1—Hg1—I1	110.65 (19)
N2—Hg1—I2	107.53 (17)
N1—Hg1—I2	103.11 (19)
I1—Hg1—I2	139.97 (3)

**Fig. 1.**
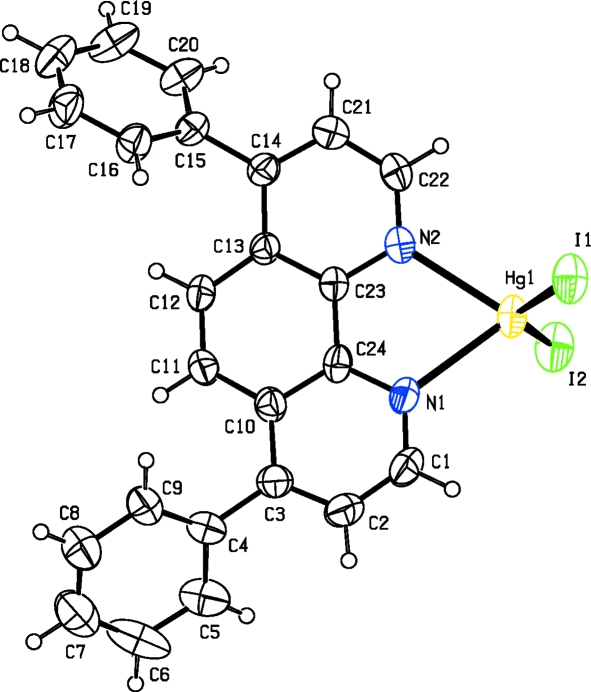
The molecular structure of the title molecule, with the atom-numbering scheme. Displacement ellipsoids are drawn at the 40% probability level.

**Fig. 2.**
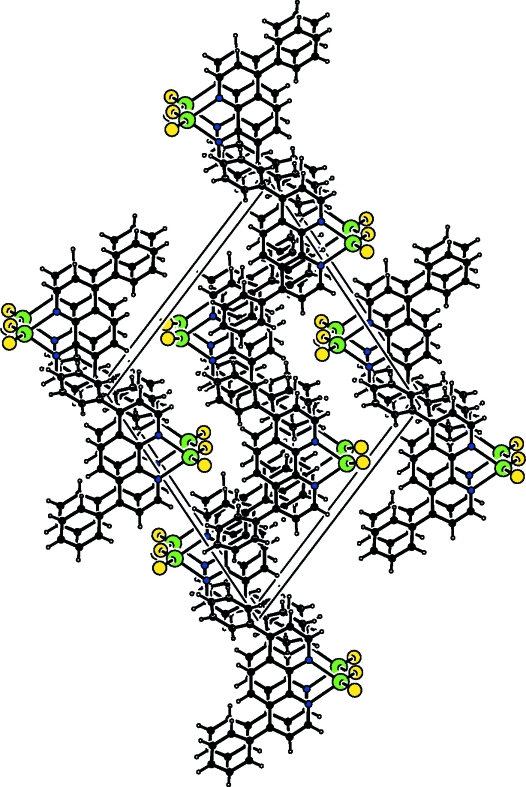
A partial packing diagram of the title compound.

## supplementary crystallographic information

### Comment

Recently, we reported the syntheses and crystal structures of [Zn(5,5'-dmbpy)Cl_2_], (II), (Khalighi *et al.*, 2008), [Zn(6-mbpy)Cl_2_], (III), (Ahmadi, Kalateh, Abedi *et al.*, 2008), [Cd(5,5'-dmbpy)(µ-Cl)_2_]_n_, (IV), (Ahmadi, Khalighi *et al.*, 2008), [Hg(5,5'-dmbpy)I_2_], (V), (Tadayon Pour *et al.*, 2008), [In(4,4'-dmbpy)Cl_3_(DMSO)], (VI), (Ahmadi, Kalateh, Ebadi *et al.*, 2008), [Cu(5,5'-dcbpy)(en)(H_2_O)_2_].2.5H_2_O, (VII), (Yousefi, Khalighi *et al.*, 2008), [Au(dmphen)Cl_2_][AuCl_4_], (VIII), (Ahmadi, Amani *et al.*, 2008), and {[HgCl(dm4bt)]_2_(µ-Cl)_2_}, (IX), (Khavasi *et al.*, 2008). [where 5,5'-dmbpy is 5,5'-dimethyl-2,2'-bipyridine, 6-mbpy is 6-methyl-2,2'-bipyridine, 4,4'-dmbpy is 4,4'-dimethyl-2,2'-bi- pyridine, DMSO is dimethyl sulfoxide, 5,5'-dcbpy is 2,2'-bipyridine-5,5'-di- carboxylate, en is ethylenediamine, dmphen is 4,7-diphenyl-1,10- phenanthroline and dm4bt is 2,2'-dimethyl-4,4'-bithiazole]. There are several Hg^II^ complexes, with formula, [HgI_2_(N—N)], such as [HgI_2_(bipy)], (X), [HgI_2_(phen)], (XI) and [HgI_2_(2,9-dmphen)], (XII), (Freire *et al.*, 1999), [HgI_2_(bipy)][HgI_2_], (XIII), (Chen *et al.*, 2006), [HgI_2_(4,4'-dmbpy)], (XIV), (Yousefi, Tadayon Pour *et al.*, 2008) and [HgI_2_(TMDA)], (XV), (Htoon & Ladd, 1976) [where bipy is 2,2'-bipyridine, phen is 1,10-phenanthroline, dmphen is 2,9-dimethyl-1,10-phenanthroline and TMDA is tetramethylethylenediamine] have been synthesized and characterized by single crystal X-ray diffraction methods. We report herein the synthesis and crystal structure of the title compound, (I).

In the title compound, (Fig. 1), the Hg^II^ atom is four-coordinated in a distorted tetrahedral configuration by two N atoms from 4,7-diphenyl-1,10-phenanthroline and two I atoms. The Hg—I and Hg—N bond lengths and angles (Table 1) are within normal ranges, as in (X), (XI) and (XIV).

In the crystal structure, the π–π contact (Fig. 2) between the pyridine and phenyl rings, Cg3···Cg4^i^ [symmetry code: (i) 3/2 - x, -1/2 + y, 1/2 - z, where Cg3 and Cg4 are centroids of the rings (N1/C1–C3/C10/C24) and (C4–C9), respectively] may stabilize the structure, with centroid–centroid distance of 4.2387 (4) Å.

### Experimental

For the preparation of the title compound, a solution of 4,7-diphenyl-1,10-phenanthroline (0.36 g, 1.10 mmol) in acetonitrile (20 ml) was added to a solution of HgI_2_ (0.50 g, 1.10 mmol) in methanol (20 ml) and the resulting colorless solution was stirred for 20 min at 313 K. This solution was left to evaporate slowly at room temperature. After one week, colorless block crystals of the title compound were isolated (yield; 0.62 g, 71.6%).

### Refinement

H atoms were positioned geometrically, with C—H = 0.93 Å for aromatic H and constrained to ride on their parent atoms with U_iso_(H) = 1.2U_eq_(C).

### Crystal data

**Table:**

[HgI_2_(C_24_H_16_N_2_)]	*F*(000) = 1440
*M**_r_* = 786.78	*D*_x_ = 2.218 Mg m^−^^3^
Monoclinic, *P*2_1_/*n*	Mo *K*α radiation, λ = 0.71073 Å
Hall symbol: -P 2yn	Cell parameters from 2356 reflections
*a* = 16.673 (3) Å	θ = 2.1–29.3°
*b* = 8.8964 (18) Å	µ = 9.17 mm^−^^1^
*c* = 16.823 (3) Å	*T* = 298 K
β = 109.26 (3)°	Block, colourless
*V* = 2355.7 (9) Å^3^	0.50 × 0.48 × 0.28 mm
*Z* = 4	

### Data collection

**Table:**

Bruker SMART CCD area-detector diffractometer	6340 independent reflections
Radiation source: fine-focus sealed tube	5356 reflections with *I* > 2σ(*I*)
graphite	*R*_int_ = 0.094
φ and ω scans	θ_max_ = 29.3°, θ_min_ = 2.1°
Absorption correction: numerical shape of crystal determined optically	*h* = −22→19
*T*_min_ = 0.016, *T*_max_ = 0.080	*k* = −12→12
19129 measured reflections	*l* = −22→23

### Refinement

**Table:**

Refinement on *F*^2^	Secondary atom site location: difference Fourier map
Least-squares matrix: full	Hydrogen site location: inferred from neighbouring sites
*R*[*F*^2^ > 2σ(*F*^2^)] = 0.067	H-atom parameters constrained
*wR*(*F*^2^) = 0.183	*w* = 1/[σ^2^(*F*_o_^2^) + (0.085*P*)^2^ + 4.523*P*] where *P* = (*F*_o_^2^ + 2*F*_c_^2^)/3
*S* = 1.23	(Δ/σ)_max_ = 0.009
6340 reflections	Δρ_max_ = 1.49 e Å^−^^3^
263 parameters	Δρ_min_ = −1.11 e Å^−^^3^
0 restraints	Extinction correction: SHELXTL (Sheldrick, 2008), Fc^*^=kFc[1+0.001xFc^2^λ^3^/sin(2θ)]^-1/4^
Primary atom site location: structure-invariant direct methods	Extinction coefficient: 0.0086 (6)

### Special details

**Table:**

Geometry. All e.s.d.'s (except the e.s.d. in the dihedral angle between two l.s. planes) are estimated using the full covariance matrix. The cell e.s.d.'s are taken into account individually in the estimation of e.s.d.'s in distances, angles and torsion angles; correlations between e.s.d.'s in cell parameters are only used when they are defined by crystal symmetry. An approximate (isotropic) treatment of cell e.s.d.'s is used for estimating e.s.d.'s involving l.s. planes.
Refinement. Refinement of *F*^2^ against ALL reflections. The weighted *R*-factor *wR* and goodness of fit *S* are based on *F*^2^, conventional *R*-factors *R* are based on *F*, with *F* set to zero for negative *F*^2^. The threshold expression of *F*^2^ > σ(*F*^2^) is used only for calculating *R*-factors(gt) *etc*. and is not relevant to the choice of reflections for refinement. *R*-factors based on *F*^2^ are statistically about twice as large as those based on *F*, and *R*- factors based on ALL data will be even larger.

### Fractional atomic coordinates and isotropic or equivalent isotropic displacement parameters (Å^2^)

**Table:**

	*x*	*y*	*z*	*U*_iso_*/*U*_eq_	
Hg1	0.35746 (2)	0.27882 (4)	0.10505 (2)	0.05561 (16)	
I1	0.29465 (5)	0.55290 (7)	0.06892 (4)	0.0694 (2)	
I2	0.32177 (5)	−0.00283 (7)	0.05316 (5)	0.0736 (2)	
N1	0.5115 (5)	0.2845 (9)	0.1529 (4)	0.0545 (17)	
N2	0.4136 (4)	0.2750 (8)	0.2559 (5)	0.0495 (14)	
C1	0.5583 (7)	0.2946 (13)	0.1039 (6)	0.065 (2)	
H1	0.5314	0.3098	0.0466	0.078*	
C2	0.6448 (7)	0.2837 (13)	0.1335 (6)	0.068 (3)	
H2	0.6749	0.2930	0.0960	0.081*	
C3	0.6884 (6)	0.2594 (10)	0.2172 (6)	0.0524 (18)	
C4	0.7831 (6)	0.2515 (11)	0.2502 (7)	0.0550 (19)	
C5	0.8260 (8)	0.1623 (13)	0.2086 (9)	0.076 (3)	
H5	0.7963	0.1118	0.1592	0.091*	
C6	0.9151 (9)	0.1506 (14)	0.2430 (12)	0.101 (5)	
H6	0.9442	0.0869	0.2182	0.122*	
C7	0.9601 (9)	0.2342 (18)	0.3143 (12)	0.093 (4)	
H7	1.0190	0.2274	0.3362	0.112*	
C8	0.9178 (7)	0.3244 (19)	0.3510 (9)	0.086 (3)	
H8	0.9477	0.3815	0.3976	0.103*	
C9	0.8304 (6)	0.3327 (15)	0.3199 (7)	0.068 (2)	
H9	0.8024	0.3948	0.3467	0.082*	
C10	0.6399 (5)	0.2429 (10)	0.2719 (5)	0.0487 (16)	
C11	0.6750 (5)	0.2082 (11)	0.3592 (6)	0.0545 (19)	
H11	0.7324	0.1843	0.3816	0.065*	
C12	0.6274 (5)	0.2090 (11)	0.4100 (5)	0.0534 (19)	
H12	0.6527	0.1855	0.4667	0.064*	
C13	0.5399 (5)	0.2448 (8)	0.3793 (5)	0.0411 (14)	
C14	0.4883 (5)	0.2524 (9)	0.4320 (5)	0.0456 (15)	
C15	0.5272 (6)	0.2456 (9)	0.5252 (5)	0.0478 (16)	
C16	0.5965 (6)	0.3362 (12)	0.5672 (5)	0.059 (2)	
H16	0.6176	0.4041	0.5370	0.071*	
C17	0.6343 (7)	0.3255 (15)	0.6545 (6)	0.071 (3)	
H17	0.6798	0.3875	0.6824	0.085*	
C18	0.6042 (9)	0.2228 (14)	0.6993 (7)	0.078 (3)	
H18	0.6300	0.2140	0.7573	0.094*	
C19	0.5350 (9)	0.1323 (12)	0.6573 (7)	0.078 (3)	
H19	0.5150	0.0622	0.6873	0.093*	
C20	0.4958 (8)	0.1460 (11)	0.5713 (6)	0.062 (2)	
H20	0.4482	0.0881	0.5441	0.075*	
C21	0.4019 (6)	0.2684 (11)	0.3937 (6)	0.058 (2)	
H21	0.3664	0.2743	0.4261	0.069*	
C22	0.3682 (5)	0.2756 (11)	0.3070 (6)	0.058 (2)	
H22	0.3094	0.2814	0.2830	0.070*	
C23	0.5005 (5)	0.2597 (8)	0.2918 (5)	0.0420 (14)	
C24	0.5506 (5)	0.2610 (9)	0.2361 (5)	0.0463 (15)	

### Atomic displacement parameters (Å^2^)

**Table:**

	*U*^11^	*U*^22^	*U*^33^	*U*^12^	*U*^13^	*U*^23^
Hg1	0.0541 (2)	0.0529 (2)	0.0461 (2)	0.00215 (13)	−0.00194 (13)	0.00031 (12)
I1	0.0728 (4)	0.0484 (3)	0.0684 (4)	0.0027 (3)	−0.0018 (3)	−0.0002 (3)
I2	0.0846 (5)	0.0496 (3)	0.0714 (4)	0.0056 (3)	0.0051 (3)	−0.0066 (3)
N1	0.049 (3)	0.072 (5)	0.036 (3)	−0.006 (3)	0.006 (3)	−0.002 (3)
N2	0.041 (3)	0.054 (4)	0.047 (3)	0.001 (3)	0.006 (3)	0.001 (3)
C1	0.071 (6)	0.084 (7)	0.037 (4)	−0.001 (5)	0.012 (4)	0.006 (4)
C2	0.066 (6)	0.093 (8)	0.051 (5)	−0.011 (5)	0.028 (4)	−0.006 (5)
C3	0.052 (4)	0.057 (4)	0.052 (4)	−0.005 (4)	0.022 (4)	−0.006 (4)
C4	0.051 (4)	0.055 (4)	0.066 (5)	0.000 (3)	0.029 (4)	0.000 (4)
C5	0.080 (7)	0.059 (5)	0.104 (8)	−0.012 (5)	0.051 (6)	−0.017 (6)
C6	0.086 (8)	0.065 (6)	0.188 (16)	0.005 (6)	0.093 (10)	0.005 (9)
C7	0.060 (6)	0.107 (10)	0.113 (11)	0.008 (7)	0.028 (7)	0.028 (9)
C8	0.057 (5)	0.111 (10)	0.085 (8)	−0.001 (7)	0.017 (5)	0.003 (8)
C9	0.047 (4)	0.085 (7)	0.069 (6)	−0.001 (5)	0.015 (4)	−0.006 (5)
C10	0.042 (3)	0.052 (4)	0.048 (4)	−0.004 (3)	0.010 (3)	−0.002 (3)
C11	0.038 (3)	0.073 (6)	0.049 (4)	0.005 (3)	0.009 (3)	0.010 (4)
C12	0.045 (4)	0.071 (5)	0.037 (3)	0.002 (4)	0.004 (3)	0.007 (3)
C13	0.040 (3)	0.045 (3)	0.037 (3)	−0.004 (3)	0.011 (3)	0.006 (3)
C14	0.049 (4)	0.047 (3)	0.041 (3)	−0.001 (3)	0.015 (3)	0.002 (3)
C15	0.057 (4)	0.047 (4)	0.040 (4)	0.000 (3)	0.016 (3)	−0.004 (3)
C16	0.063 (5)	0.063 (5)	0.048 (4)	−0.011 (4)	0.013 (4)	−0.001 (4)
C17	0.074 (6)	0.079 (6)	0.050 (5)	−0.003 (5)	0.005 (4)	−0.019 (5)
C18	0.104 (9)	0.087 (8)	0.040 (4)	0.004 (7)	0.021 (5)	0.001 (5)
C19	0.128 (10)	0.060 (5)	0.058 (5)	−0.010 (6)	0.048 (6)	−0.008 (5)
C20	0.091 (7)	0.051 (4)	0.052 (4)	−0.006 (4)	0.033 (4)	−0.004 (4)
C21	0.050 (4)	0.070 (5)	0.057 (5)	0.000 (4)	0.022 (4)	−0.003 (4)
C22	0.039 (4)	0.072 (6)	0.057 (5)	−0.006 (4)	0.008 (3)	−0.003 (4)
C23	0.040 (3)	0.042 (3)	0.040 (3)	−0.001 (3)	0.007 (3)	−0.001 (3)
C24	0.047 (4)	0.044 (3)	0.039 (3)	−0.002 (3)	0.003 (3)	0.007 (3)

### Geometric parameters (Å, °)

**Table:**

I1—Hg1	2.6441 (8)	C11—H11	0.9300
I2—Hg1	2.6555 (9)	C12—C13	1.415 (11)
N1—Hg1	2.425 (7)	C12—H12	0.9300
N2—Hg1	2.399 (7)	C13—C23	1.407 (10)
C1—N1	1.312 (12)	C13—C14	1.426 (10)
C1—C2	1.364 (15)	C14—C21	1.379 (12)
C1—H1	0.9300	C14—C15	1.487 (11)
C2—C3	1.372 (14)	C15—C20	1.389 (12)
C2—H2	0.9300	C15—C16	1.394 (12)
C3—C10	1.420 (12)	C16—C17	1.398 (13)
C3—C4	1.493 (13)	C16—H16	0.9300
C4—C9	1.382 (15)	C17—C18	1.380 (18)
C4—C5	1.401 (14)	C17—H17	0.9300
C5—C6	1.409 (19)	C18—C19	1.393 (18)
C5—H5	0.9300	C18—H18	0.9300
C6—C7	1.40 (2)	C19—C20	1.383 (15)
C6—H6	0.9300	C19—H19	0.9300
C7—C8	1.34 (2)	C20—H20	0.9300
C7—H7	0.9300	C21—C22	1.381 (14)
C8—C9	1.378 (15)	C21—H21	0.9300
C8—H8	0.9300	C22—N2	1.320 (12)
C9—H9	0.9300	C22—H22	0.9300
C10—C24	1.419 (11)	C23—N2	1.379 (10)
C10—C11	1.423 (12)	C23—C24	1.447 (11)
C11—C12	1.345 (13)	C24—N1	1.352 (10)
			
N2—Hg1—N1	69.4 (2)	C12—C11—C10	121.7 (8)
N2—Hg1—I1	104.08 (17)	C12—C11—H11	119.1
N1—Hg1—I1	110.65 (19)	C10—C11—H11	119.1
N2—Hg1—I2	107.53 (17)	C11—C12—C13	121.5 (7)
N1—Hg1—I2	103.11 (19)	C11—C12—H12	119.2
I1—Hg1—I2	139.97 (3)	C13—C12—H12	119.2
C1—N1—C24	118.5 (8)	C23—C13—C12	118.2 (7)
C1—N1—Hg1	125.3 (6)	C23—C13—C14	118.5 (7)
C24—N1—Hg1	115.9 (6)	C12—C13—C14	123.0 (7)
C22—N2—C23	117.4 (7)	C21—C14—C13	117.8 (7)
C22—N2—Hg1	125.6 (6)	C21—C14—C15	121.5 (7)
C23—N2—Hg1	116.8 (5)	C13—C14—C15	120.7 (7)
N1—C1—C2	122.9 (8)	C20—C15—C16	119.2 (8)
N1—C1—H1	118.6	C20—C15—C14	120.0 (8)
C2—C1—H1	118.6	C16—C15—C14	120.8 (8)
C1—C2—C3	121.6 (8)	C15—C16—C17	120.3 (9)
C1—C2—H2	119.2	C15—C16—H16	119.8
C3—C2—H2	119.2	C17—C16—H16	119.8
C2—C3—C10	117.4 (8)	C18—C17—C16	120.0 (11)
C2—C3—C4	121.8 (8)	C18—C17—H17	120.0
C10—C3—C4	120.8 (8)	C16—C17—H17	120.0
C9—C4—C5	118.6 (9)	C17—C18—C19	119.7 (10)
C9—C4—C3	121.9 (8)	C17—C18—H18	120.2
C5—C4—C3	119.5 (9)	C19—C18—H18	120.2
C4—C5—C6	118.6 (12)	C20—C19—C18	120.4 (10)
C4—C5—H5	120.7	C20—C19—H19	119.8
C6—C5—H5	120.7	C18—C19—H19	119.8
C7—C6—C5	120.5 (11)	C19—C20—C15	120.3 (10)
C7—C6—H6	119.8	C19—C20—H20	119.8
C5—C6—H6	119.8	C15—C20—H20	119.8
C8—C7—C6	119.7 (12)	C14—C21—C22	119.8 (8)
C8—C7—H7	120.1	C14—C21—H21	120.1
C6—C7—H7	120.1	C22—C21—H21	120.1
C7—C8—C9	120.5 (14)	N2—C22—C21	124.5 (8)
C7—C8—H8	119.8	N2—C22—H22	117.7
C9—C8—H8	119.8	C21—C22—H22	117.7
C8—C9—C4	122.0 (11)	N2—C23—C13	121.9 (7)
C8—C9—H9	119.0	N2—C23—C24	117.6 (7)
C4—C9—H9	119.0	C13—C23—C24	120.5 (7)
C24—C10—C3	117.0 (8)	N1—C24—C10	122.6 (8)
C24—C10—C11	118.7 (7)	N1—C24—C23	119.2 (7)
C3—C10—C11	124.2 (8)	C10—C24—C23	118.2 (7)
			
C1—N1—Hg1—N2	−177.4 (9)	C21—C14—C15—C20	51.1 (12)
C24—N1—Hg1—N2	9.2 (6)	C13—C14—C15—C20	−129.9 (9)
C1—N1—Hg1—I1	−79.6 (9)	C21—C14—C15—C16	−130.2 (10)
C24—N1—Hg1—I1	107.0 (6)	C13—C14—C15—C16	48.7 (12)
C1—N1—Hg1—I2	78.6 (9)	C20—C15—C16—C17	0.7 (15)
C24—N1—Hg1—I2	−94.9 (6)	C14—C15—C16—C17	−177.9 (9)
C22—N2—Hg1—N1	177.5 (8)	C15—C16—C17—C18	1.3 (17)
C23—N2—Hg1—N1	−7.5 (5)	C16—C17—C18—C19	−1.3 (19)
C22—N2—Hg1—I1	70.4 (8)	C17—C18—C19—C20	−0.7 (19)
C23—N2—Hg1—I1	−114.6 (5)	C18—C19—C20—C15	2.8 (17)
C22—N2—Hg1—I2	−84.7 (7)	C16—C15—C20—C19	−2.7 (15)
C23—N2—Hg1—I2	90.3 (6)	C14—C15—C20—C19	175.9 (9)
N1—C1—C2—C3	0.8 (19)	C13—C14—C21—C22	0.4 (14)
C1—C2—C3—C10	1.2 (16)	C15—C14—C21—C22	179.4 (9)
C1—C2—C3—C4	−178.3 (10)	C14—C21—C22—N2	−3.1 (16)
C2—C3—C4—C9	130.8 (11)	C12—C13—C23—N2	171.4 (8)
C10—C3—C4—C9	−48.7 (14)	C14—C13—C23—N2	−2.7 (11)
C2—C3—C4—C5	−47.7 (14)	C12—C13—C23—C24	−9.3 (11)
C10—C3—C4—C5	132.8 (10)	C14—C13—C23—C24	176.6 (7)
C9—C4—C5—C6	4.5 (17)	C3—C10—C24—N1	3.7 (13)
C3—C4—C5—C6	−177.0 (10)	C11—C10—C24—N1	−175.7 (8)
C4—C5—C6—C7	−4(2)	C3—C10—C24—C23	−174.0 (7)
C5—C6—C7—C8	1(2)	C11—C10—C24—C23	6.7 (12)
C6—C7—C8—C9	1(2)	N2—C23—C24—N1	3.3 (11)
C7—C8—C9—C4	−1(2)	C13—C23—C24—N1	−176.0 (7)
C5—C4—C9—C8	−2.2 (18)	N2—C23—C24—C10	−179.0 (7)
C3—C4—C9—C8	179.3 (12)	C13—C23—C24—C10	1.7 (11)
C2—C3—C10—C24	−3.3 (13)	C21—C22—N2—C23	2.8 (14)
C4—C3—C10—C24	176.3 (8)	C21—C22—N2—Hg1	177.8 (7)
C2—C3—C10—C11	176.0 (9)	C13—C23—N2—C22	0.2 (12)
C4—C3—C10—C11	−4.4 (14)	C24—C23—N2—C22	−179.2 (8)
C24—C10—C11—C12	−7.6 (14)	C13—C23—N2—Hg1	−175.2 (6)
C3—C10—C11—C12	173.1 (9)	C24—C23—N2—Hg1	5.5 (9)
C10—C11—C12—C13	−0.1 (15)	C2—C1—N1—C24	−0.6 (16)
C11—C12—C13—C23	8.6 (13)	C2—C1—N1—Hg1	−173.9 (9)
C11—C12—C13—C14	−177.5 (9)	C10—C24—N1—C1	−1.7 (14)
C23—C13—C14—C21	2.3 (12)	C23—C24—N1—C1	175.9 (9)
C12—C13—C14—C21	−171.5 (8)	C10—C24—N1—Hg1	172.2 (6)
C23—C13—C14—C15	−176.7 (7)	C23—C24—N1—Hg1	−10.2 (10)
C12—C13—C14—C15	9.5 (12)		
